# Comparative Transcriptome Analysis Reveals the Cause for Accumulation of Reactive Oxygen Species During Pollen Abortion in Cytoplasmic Male-Sterile Kenaf Line 722HA

**DOI:** 10.3390/ijms20215515

**Published:** 2019-11-05

**Authors:** Bujin Zhou, Yiding Liu, Zhengxia Chen, Dongmei Liu, Yining Wang, Jie Zheng, Xiaofang Liao, Ruiyang Zhou

**Affiliations:** 1 College of Life Science and Technology, Guangxi University, Nanning 530004, Guangxi, China; zhou_bujin@163.com; 2 College of Agriculture, Guangxi University, Nanning 530004, Guangxi, China; liuyiding1988@163.com (Y.L.);; 3 Department of Life Science and Food, Shangqiu Normal University, Shangqiu 476000, Henan, China; 4 Cash Crop Institute of Guangxi Academy of Agricultural Sciences, Nanning 530007, Guangxi, China

**Keywords:** kenaf, cytoplasmic male sterility (CMS), transcriptome, reactive oxygen species (ROS), microspore development

## Abstract

Cytoplasmic male sterility (CMS) is a maternally inherited trait used for hybrid production in plants, a novel kenaf CMS line 722HA was derived from the thermo-sensitive male-sterile mutant ‘HMS’ by recurrent backcrossing with 722HB. The line 722HA has great potential for hybrid breeding in kenaf. However, the underlying molecular mechanism that controls pollen abortion in 722HA remains unclear, thus limiting the full utilization of this line. To understand the possible mechanism governing pollen abortion in 722HA, cytological, transcriptomic, and biochemical analyses were carried out to compare the CMS line 722HA and its maintainer line 722HB. Cytological observations of the microspore development revealed premature degradation of the tapetum at the mononuclear stage, which resulted in pollen dysfunction. The k-means clustering analysis of differentially expressed genes (DEGs) revealed that these genes are related to processes associated with the accumulation of reactive oxygen species (ROS), including electron transport chain, F_1_F_0_-ATPase proton transport, positive regulation of superoxide dismutase (SOD), hydrogen peroxide catabolic, and oxidation-reduction. Biochemical analysis indicated that ROS-scavenging capability was lower in 722HA than in 722HB, resulting in an accumulation of excess ROS, which is consistent with the transcriptome results. Taken together, these results demonstrate that excessive ROS accumulation may affect the normal development of microspores. Our study provides new insight into the molecular mechanism of pollen abortion in 722HA and will promote further studies of kenaf hybrids.

## 1. Introduction

Cytoplasmic male sterility (CMS), in which a maternally inherited trait causes dysfunctional pollen, is a natural phenomenon that widely exists among higher plants. CMS has been identified and characterized in approximately 153 angiosperm taxa [1]. The discovery and utilization of plant CMS have produced excellent germplasm resources for increase in crop yield, improvement of quality, and disease resistance. To date, the CMS system has been used in the breeding of various crops, including rice, corn, rapeseed, and cotton [2,3,4]. 

Kenaf (*Hibiscus cannabinus* L.) is an important multi-use crop of the family Malvaceae and produces both fibre and oil. Due to its high hygroscopicity, gas permeability, and antistatic properties, kenaf is widely used in the paper pulp and textile industries [5,6] for products such as high-grade fabrics, filler, cloth, carpet, automobile components, and furniture. The seeds of kenaf are high in monounsaturated and polyunsaturated fatty acids (PUFAs) [7] and can be used as industrial materials, e.g., in cosmetics and industrial lubricants, and for biofuel production. The discovery and utilization of cytoplasmic male-sterile kenaf plants can significantly improve yield, quality, and resistance, supporting the practical value of kenaf breeding [8,9,10]. In the past few decades, several natural cytoplasmic male-sterile kenaf mutants have been found [11,12,13] and have been used for the production of kenaf three-line hybrids. Furthermore, a novel transgenic kenaf male-sterile mutant, ‘HMS’, was found in an experimental field by our group, and a stable kenaf CMS line, 722HA, which has great potential for breeding F1 hybrids, was developed via recurrent backcrossing to 722HB. Nevertheless, to date, the molecular regulatory mechanism controlling pollen abortion remains unknown, which has limited the widespread use of kenaf CMS line 722HA in hybrid production, so far.

Anther development is a programmed process of stamen maturation during which functional microspores or pollen grains are produced for propagation; this process is a critical phase in the plant life cycle, and a series of genes regulate this complex biological process [14]. These genes are mainly involved in different biological pathways, including reactive oxygen species ROS metabolism [15,16] energy metabolism [17,18], lipid metabolism [19], carbohydrate metabolism [17,20], signal transduction [21], etc. Abnormal expression of these genes can lead to male sterility [22]. Thus, understanding the gene expression pattern during anther development is useful for elucidating the molecular regulatory mechanisms of pollen abortion in plant CMS.

As a comprehensive and rapid technology, RNA sequencing (RNA-seq) can be used to obtain nearly the entire transcriptional information concerning gene expression in tissues and organs. This technology has been widely used in gene discovery, functional identification, and genetic improvements. In the past few years, to elucidate the regulatory mechanism of plant CMS, transcriptome analyses have been conducted in many higher plant species, including soybean, sesame, cabbage, and cotton [14,16,23,24]. In addition, numerous differentially expressed genes (DEGs) involved in pollen dysfunction have been identified in kenaf CMS line P3A via comparative transcriptome analysis [25]. Most of these genes are associated with anther development, energy metabolism, transcription factors (TFs) (MYB and MADS-box TFs), chaperones, and fertility restoration. In contrast to these findings, the gene expression patterns during anther development in kenaf CMS line 722HA are poorly understood.

In the present study, the CMS line 722HA was investigated in comparison to its maintainer line 722HB to understand the molecular mechanism of CMS in 722HA and to explore the different biological processes involved in pollen abortion as well as the cytological characteristics of the anthers during pollen development. An RNA-seq-based transcriptome analysis of anther development at four developmental stages (tetrad, mononuclear, dinuclear, and mature pollen grain stage) in 722HA and 722HB was subsequently carried out, and the expression patterns of genes involved in anther development were determined to identify potential candidate genes responsible for pollen abortion in 722HA. These results will provide a preliminary understanding of the molecular mechanism of pollen abortion in 722HA and facilitate the breeding of kenaf hybrids.

## 2. Results

### 2.1. Morphological and Cytological Characterization of 722HA and 722HB

**Figure 1:**
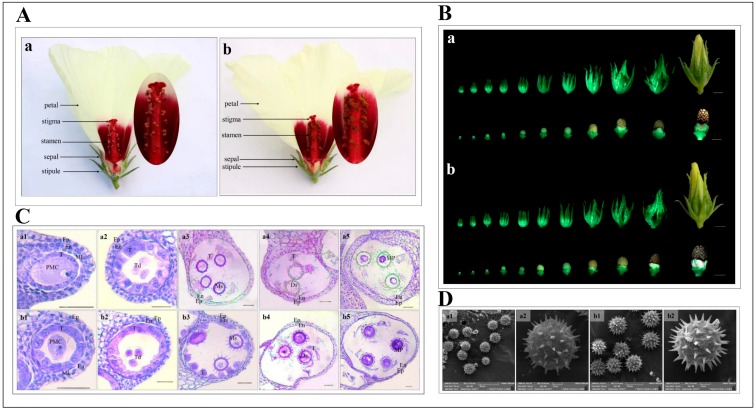
Phenotypic and cytological characterization of kenaf flowers. (**A**) Morphological characterization of kenaf flowers of the Cytoplasmic male sterility (CMS) line 722HA and its maintainer line 722HB. (**B**) Phenotypic characterization of kenaf flower buds and anther of 722HA and 722HB. a: Phenotypes of the 722HA flower buds and anther; b: Phenotypes of the 722HB flower buds and anther; Bar = 0.5 cm. (**C**) Microspore development of the kenaf anther of 722HA (a1–a5) and 722HB (b1–b5). a1, b1; a2, b2; a3, b3; a4, b4, and a5, b5 correspond to microspores of pollen mother cell, tetrad, mononuclear, dinuclear, and mature pollen grains stages, respectively. PMC: Pollen mother cell, Ep: Epidermis; En: Endothecium; ML: Middle layer; T: Tapetum; Td: Tetrad microspore; Ms: Mononuclear microspore; Ds: Dinuclear microspore; MP: Mature pollen grain; Bar = 20 μm. (**D**) The pollen characterization via SEM. a1, a2: SEM-based structure of the pollen in 722HA. b1, b2: SEM-based structure of the pollen in 722HB.

At the flower development stage, the morphological characteristics of the flowers were observed and compared between kenaf CMS lines 722HA and its maintainer line 722HB. There were no obvious differences in the morphology of the petals, stigma, sepals, and stipules between 722HA and 722HB (Figure 1A). The anthers of 722HB were typically purple, and mature pollen grains were released from the dehisced pollen sacs (Figure 1A,B). However, the anthers of 722HA were plump but not dehiscent (Figure 1A,B).

To understand the timing and characteristics of pollen abortion in the kenaf CMS line 722HA, the anthers at different developmental stages were cytologically characterized via paraffin sectioning. During the pollen mother cell stage and the tetrad stage, 722HA and 722HB developed normally, and the pollen sac of each consisted of tapetum, middle layer, endothelial layer, epidermis, and pollen mother cells (Figure 1C). The pollen mother cells entered the tetrad stage after meiosis. At the mononuclear stage, the microspore cell nuclei in both 722HA and 722HB were gradually pressed to the cell edge by the large central vacuole, and the tapetum began to degrade. However, the degradation rate of the tapetal cells of 722HA at the mononuclear stage was significantly faster than that of 722HB (Figure 1C). At the dinuclear stage, the microspores of 722HB contained an abundance of content, providing sufficient nutrients for pollen development. However, the content of the microspores of 722HA was significantly less than that of 722HB (Figure 1C). During the mature pollen grain stage, all the microspores of 722HB became mature pollen grains and contained an abundance of nutrients. In contrast, the microspores of 722HA showed signs of plasmolysis and lacked nutrients (Figure 1C). Cytological observations also revealed that the microspores of 722HA developed into aborted pollen grains because of the nutrient deficiency caused by the premature degradation of the tapetal cells. In addition, the morphology of the pollen was characterized via scanning electron microscopy (SEM). Except for the smaller spines of the pollen of 722HA, there were no significant differences in pollen size between 722HA and 722HB (Figure 1D).

### 2.2. De Novo Assembly and Sequence Annotation

To further understand the molecular mechanisms of pollen abortion in 722HA, RNA-seq was performed via the BGI-500 sequencing platform. After the raw data were trimmed, a total of 1.125 billion clean reads for the CMS line samples and 1.129 billion clean reads for the fertile samples were obtained, and 97.67% and 91.00% of the bases among the clean reads had quality values greater than 20 (Q20) and 30 (Q30), respectively (Table 1, Table S1). In total, 2.25 billion clean reads were assembled using the Trinity program, and 2,720,278 transcripts were obtained with a mean length of 933 nt. TGICL was used to cluster the assembled transcripts to reduce their numbers. We obtained 228,199 unigenes whose average length was 1839 nt and whose N50 was 2565 nt (Table S1).

**Figure 2:**
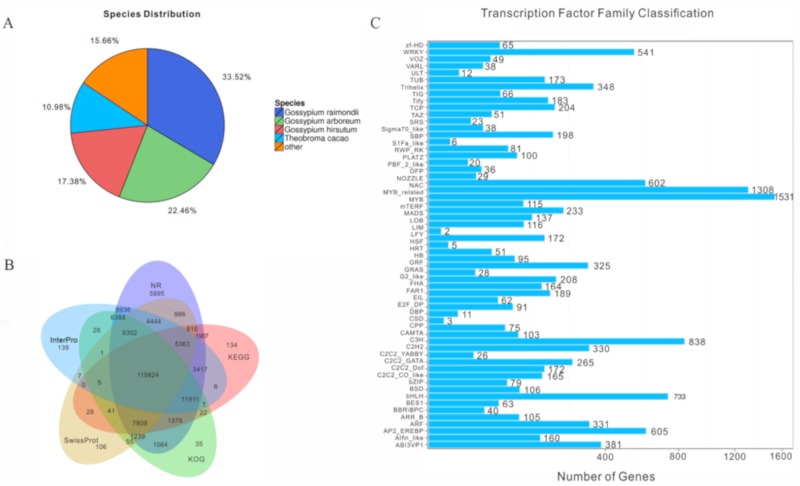
Functional annotation of kenaf unigenes. (**A**) The distribution of non-redundant (NR) annotated species is shown as a percentage of the total homologous sequence. (**B**) Venn diagram comprising the results of the NR, EuKaryotic Orthologous Groups (KOG), Kyoto Encyclopedia of Genes and Genomes (KEGG), Swiss-Prot, and InterPro database information. (**C**) Transcription factor (TF) family classification of unigenes. The X axis represents the number of unigenes. The Y axis represents the TF family.

On the basis of the non-redundant (NR) database functional annotation results, the percentages of different species in the unigene annotations were calculated. The species distribution was as follows: 33.52% of the distinct sequences had top matches (first hit) with sequences from *Gossypium raimondii* (22.46%), followed by *Gossypium arboreum* (22.46%), *Gossypium hirsutum* (17.38%), *Theobroma cacao* (10.98%), *Herrania umbratica* (6.44%), *Corchorus olitorius* (2.08%), and *Corchorus capsularis* (1.98%) (Figure 2A). The clustered unigenes were then subjected to functional annotation against seven databases (NR, NT, GO, KOG, KEGG, Swiss-Prot, and InterPro), which yielded 186,777 (NR: 81.85%), 187,071 (NT: 81.98%), 146,026 (Swiss-Prot: 63.99%), 148,660 (KEGG: 65.14%), 155,101 (KOG: 67.97%), 165,772 (InterPro: 72.64%), and 126,441 (GO: 55.41%) annotations (Figure 2B). After annotation, unigenes that encoded TFs were predicted, and TF families were classified. The classification of the TF families showed that the MYB family (1533 genes; 12.48%) was predicted to be the largest TF family (Figure 2C; Table S2), followed by the MYB-related (1308 genes; 10.64%), C3H family (838 genes, 6.82%), bHLH family (733 genes; 5.97%), AP2-EREBP family (605, 4.92%), NAC family (602 genes; 4.90%), etc. (Figure 2C).

On the basis of the GO classification and functional enrichment, a total of 228,199 unigenes were classified into three main independent categories (cell components, molecular functions, and biological processes) and 60 sub-categories (Figure S1). In the cellular component category, membrane, membrane part, cell, cell part, and organelle were the important sub-categories. In the molecular function category, binding and catalytic activity were the primary sub-categories. In the biological process category, the top two sub-categories were cellular process and metabolic process (Figure S1).

### 2.3. Detection of Differential Gene Expression between 722HA and 722HB

**Figure 3:**
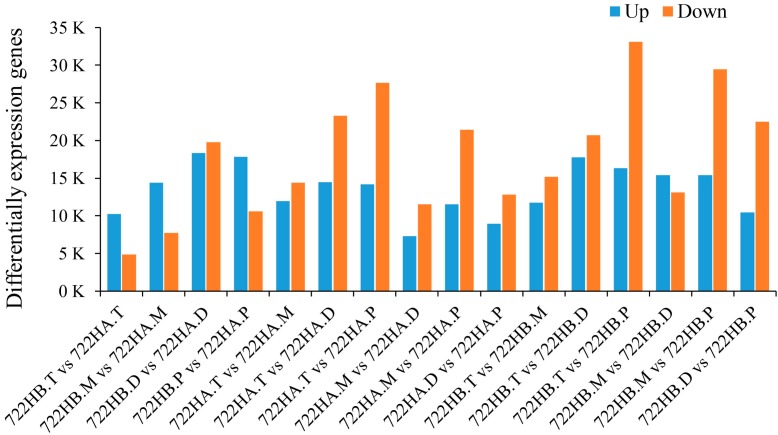
Differentially expressed gene (DEG) statistics. The X axis represents the groups. The Y axis represents the numbers of DEGs. 722HA.T, 722HA.M, 722HA.D, and 722HA.P correspond to microspores of the tetrad, mononuclear, dinuclear, and mature pollen grains stages, respectively, in 722HA; 722HB.T, 722HB.M, 722HB.D, and 722HB.P correspond to microspores of the tetrad, mononuclear, dinuclear, and mature pollen grain stages, respectively, in 722HB.

Fragments per kilobase of transcript per million base pairs sequenced (FPKM) values were calculated for all samples on the basis of the assembled transcript sequences. Total of 207,047 transcripts (FPKM > 0) and 206,399 transcripts (FPKM > 1) were found in at least one sample; the transcript FPKM value distribution for each sample is shown in Figure S2. According to the developmental timeline, samples from the previous time point of a single species were used to compare with all subsequent time points. In addition, the DEGs between different varieties at the same time point was determined. A total of 90,007 DEGs were identified on the basis of the following criteria: A fold change ≥ 2.00 and a probability ≥ 0.8 (Figure 3). The correlation coefficients between replicate samples at a single time point were larger than 0.9 (Figure S3); therefore, the average expression levels of all replicate samples at a single time point were used as the expression level of a single time point. Among these DEGs, 34,735 and 20,930 transcripts had expression levels larger than five and 10 in at least one group. To reduce the noise impact of low-expression transcripts, only genes with expression level changes larger than 10-fold in at least one set of samples were selected for subsequent analysis.

### 2.4. Hierarchical Clustering and Principal Component Analysis (PCA)

**Figure 4:**
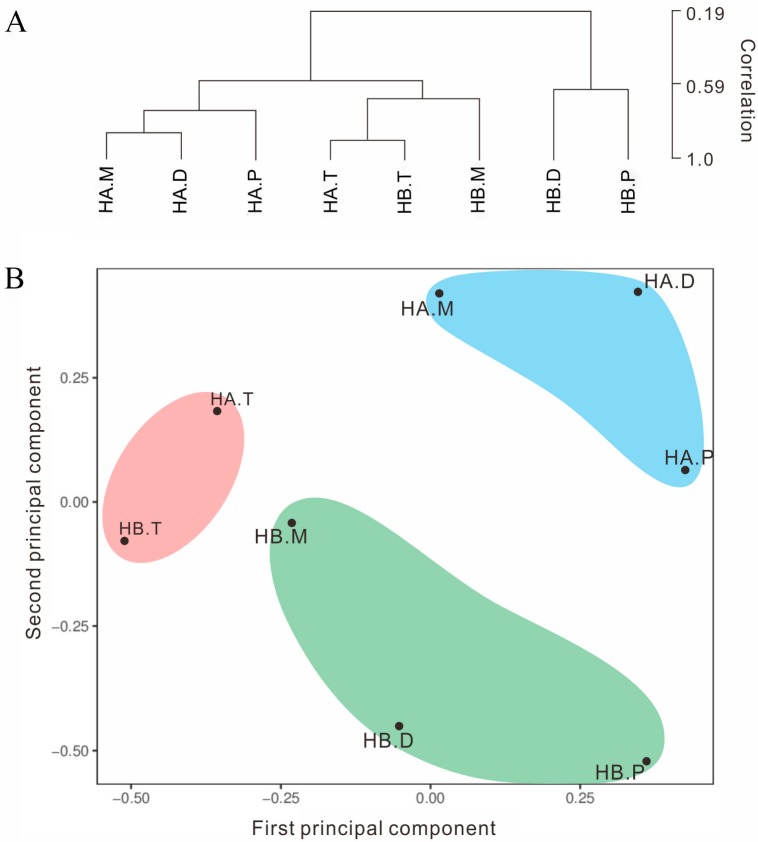
Hierarchical clustering and Principal Component Analysis (PCA). (**A**) Hierarchical clustering tree. (**B**) PCA clustering results. HA.T, HA.M, HA.D, and HA.P correspond to microspore of the tetrad, mononuclear, dinuclear, and mature pollen grains stages, respectively, in 722HA; HB.T, HB.M, BA.D, and HB.P correspond to microspores of the tetrad, mononuclear, dinuclear, and mature pollen grains stage, respectively, in 722HB.

To understand the overall transcriptome dynamics of anther development in 722HA and 722HB, hierarchical clustering and PCA of 20,930 transcripts were performed. The results showed that the expression patterns of the transcripts were very similar at the tetrad stage in 722HA and 722HB. However, the transcripts in 722HA and 722HB from the mononuclear stage onward exhibited completely different expression patterns. In 722HB, the expression patterns of the transcripts were similar at the mononuclear and tetrad stages, but the expression patterns between the dinuclear stage and mononuclear stage were significantly different, as were those between the dinuclear stage and the mature pollen grain stage. In 722HA, there were significant differences in expression characteristics between the mononuclear and tetrad stage and between the mature pollen grain stage and the dinuclear stage, whereas the expression patterns of transcripts at the dinuclear stage and mononuclear stage were not significantly different (Figure 4).

### 2.5. Gene Expression Pattern in 722HA and 722HB

**Figure 5:**
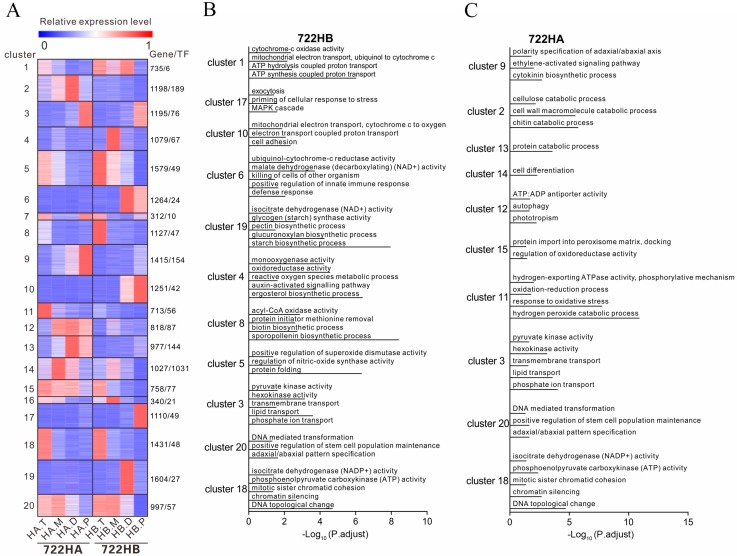
Results of K-means clustering. (**A**) Heat diagram of K-means clustering. (**B**) Gene Ontology (GO) enrichment result of the highly expressed set of genes in 722HB. (**C**) GO enrichment result of the highly expressed set of genes in 722HA. HA.T, HA.M, HA.D, and HA.P correspond to microspores of the tetrad, mononuclear, dinuclear, and mature pollen grains stage, respectively, in 722HA; HB.T, HB.M, BA.D, and HB.P correspond to microspore of the tetrad, mononuclear, dinuclear, and mature pollen grains stage, respectively, in 722HB.

To investigate the gene expression patterns and functional changes at different developmental time points, we performed k-means clustering on 20,930 transcripts (which including 1333 TFs), resulting in the clustering of 20 co-expression modules. The genes of the modules were subsequently subjected to KEGG and GO enrichment analyses, and the context likelihood of relatedness (CLR) algorithm was used to infer which TFs might be associated with target genes. Of the expression levels of the 20 co-expression modules, only three modules were similar between 722HA and 722HB (classified by developmental timeline). The first co-expression module (Cluster 18) was related to DNA topological change and mitotic sister chromatid cohesion, and the genes in Cluster 18 were highly expressed at the tetrad stage in 722HA and 722HB. These genes mainly encoded isocitrate dehydrogenase and phosphoenolpyruvate carboxykinase enzymes, and the linked TFs of Cluster 18 genes were mainly MYB, FAR1, and AP2-EREBP. The second co-expression module (Cluster 20) was related to stem cells. The genes in Cluster 20 were highly expressed at the tetrad and mononuclear stages in 722HA and 722HB, but their expression began to decrease at dinuclear and reached the lowest level at the mature pollen grain stage. The genes in Cluster 20 were enriched in adaxial/abaxial pattern specification and positive regulation of stem cell population maintenance. The linked TFs of Cluster 20 genes were mainly TUB, C3H, and C2H2. The third co-expression module (Cluster 3) was related to transmembrane transport. The genes in Cluster 3 were highly expressed at the mature pollen grain stage in 722HA and 722HB, and the expression levels of the genes in this cluster in 722HA were slightly greater than those in 722HB. Cluster 3 genes were enriched in lipid transport and transmembrane transport, and Cluster 3 genes mainly encoded the hexokinase and pyruvate kinase enzymes. The linked TFs of Cluster 3 genes were mainly Tify, bHLH, and MYB (Figure 5, Tables S3–S5).

Eight types of co-expressed genes were highly expressed in 722HB (classified by developmental timeline). The first co-expression module (Cluster 5) was related to superoxide dismutase (SOD). The genes in Cluster 5 were highly expressed only at the tetrad stage in 722HB, and the expression levels of these genes in 722HA were significantly lower than those in 722HB. The genes in Cluster 5 were related to protein folding and positive regulation of SOD activity. The linked TFs of Cluster 5 genes were mainly bHLH, Tify, and LIM. The second co-expression module (Cluster 8) was related to the pollen exine layer. The genes in Cluster 8 were highly expressed only at the tetrad stage in 722HB and were related to the sporopollenin biosynthetic process and biotin biosynthetic process. Cluster 8 genes mainly encoded acyl-CoA oxidase and biotin synthase. The linked TFs of Cluster 8 genes were mainly GeBP, MYB, and MADS. The third co-expression module (Cluster 4) was related to the cell membrane. The genes in Cluster 4 were highly expressed only at the mononuclear stage in 722HB and were related to ergosterol biosynthetic process, auxin-activated signalling pathway, cell membrane integrity, and cell membrane-bound enzyme activity. Cluster 4 genes mainly encoded oxidoreductases and monooxygenases. The linked TFs of Cluster 4 genes were mainly LOB, ARF, and MYB. The fourth co-expression module (Cluster 19) was related to the cell wall. The genes in Cluster 19 were highly expressed only at the dinuclear stage in 722HB and were related to glucuronoxylan biosynthetic processes and pectin biosynthetic processes. Cluster 19 genes mainly encoded glycogen (starch) synthase and isocitrate dehydrogenase. The linked TFs of Cluster 19 genes were mainly zf-HD, C3H, and LOB. The fifth co-expression module (Cluster 6) was related to protection mechanisms. The genes in Cluster 6 were highly expressed only at the dinuclear and mature pollen grain stages in 722HB and were related to the defence response and positive regulation of the innate immune response. Cluster 6 genes mainly encoded malate dehydrogenase and ubiquinol-cytochrome-c reductase. The linked TFs of Cluster 6 genes were mainly LOB, LIM, and C3H. The sixth co-expression module (Cluster 10) was related to oxygen transport. The genes in Cluster 10 were highly expressed only at the dinuclear and mature pollen grain stages in 722HB and were associated with cell adhesion and cytochrome-c to oxygen. The linked TFs of Cluster 10 genes were mainly MYB, MADS, and LOB. The seventh co-expression module (Cluster 17) was related to extracellular secretion. The genes in Cluster 17 were highly expressed only at the mature pollen grain stage in 722HB and were associated with the MAPK cascade and exocytosis. The linked TFs of Cluster 17 genes were mainly PLATZ, TUB, and bHLH. The eighth co-expression module (Cluster 1) was related to energy transfer. The genes in Cluster 1 were highly expressed from the tetrad to the dinuclear stage in 722HB and highly expressed only at the tetrad stage in 722HA. Cluster 1 genes were enriched in ATP hydrolysis/synthesis coupled proton transport and mitochondrial electron transport (ubiquinol to cytochrome-c). Cluster 1 contained genes encoding genes for the subunits of F_1_F_0_-ATPase and cytochrome-c oxidase. The linked TFs of Cluster 1 genes were mainly bHLH, AP2-EREBP, and C3H (Figure 5, Tables S4–S6).

Seven types of genes were highly expressed in 722HA (according to the developmental timeline). The first co-expression module (Cluster 11) was related to ROS. The genes in Cluster 11 were highly expressed only at the tetrad stage in 722HA and were related to the hydrogen peroxide catabolic process and oxidation-reduction process. The linked TFs of Cluster 11 genes were mainly MADS, ABI3VP1, and AP2-EREBP. The second co-expression module (Cluster 15) was related to oxidoreductase. The genes in Cluster 15 were continuously expressed from the tetrad stage to the dinuclear stage in 722HA and were highly expressed only at the tetrad stage in 722HB. Cluster 15 genes were enriched in the regulation of oxidoreductase activity and protein import into the peroxisome matrix. The linked TFs of Cluster 15 genes were mainly PLATZ, HSF, and GeBP. The third co-expression module (Cluster 12) was related to autophagy. The genes in Cluster 12 were continuously expressed from the mononuclear stage to mature pollen grain stage in 722HA and were related to phototropism and autophagy. Cluster 12 genes mainly encoded cysteine-type endopeptidase and ATP:ADP antiporter. The linked TFs of Cluster 12 genes were mainly GRAS, EIL, and WRKY. The fourth co-expression module (Cluster 14) was related to cell differentiation. The genes in Cluster 14 were expressed at the highest level at the mononuclear stage in 722HA and were expressed at the mononuclear stage of 722HB, albeit at a significantly lower level than in 722HA. The linked TFs of Cluster 14 genes were mainly NAC, AP2-EREBP, and GRAS. The fifth co-expression module (Cluster 13) was related to proteolysis. The genes in Cluster 13 were highly expressed only at the dinuclear and mature pollen grain stages in 722HA and were associated with protein transport to vacuole involved in ubiquitin-dependent protein catabolic process via the multivesicular body sorting pathway. The linked TFs of Cluster 13 genes were mainly BES1, NAC, GRAS, etc. The sixth co-expression module (Cluster 2) was related to catabolism. The genes in Cluster 2 were expressed at all stages in 722HA and peaked at the dinuclear stage. Cluster 2 genes were related to chitin catabolic process and cell wall macromolecule catabolic process. The linked TFs of Cluster 2 genes were mainly C2C2-GATA, NAC, and MYB. The seventh co-expression module (Cluster 9) was related to cytokinin. The genes in Cluster 9 were highly expressed only at the mature pollen grain stage in 722HA and were related to the cytokinin biosynthetic process and polarity specification of the adaxial/abaxial axis. The linked TFs of Cluster 9 genes were mainly NAC, MYB, and AP2-EREBP (Figure 5, Tables S4–S6).

### 2.6. Malondialdehyde (MDA) Content and SOD Activity Analysis

**Figure 6:**
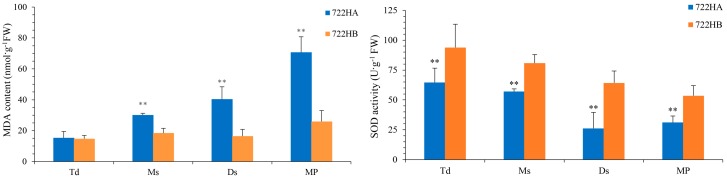
Malondialdehyde (MDA) contents and Superoxide dismutase (SOD) activity in the anthers of 722HA and 722HB at various developmental stages. Td: Tetrad stage; Ms: Mononuclear stage; Ds: Dinuclear stage; MP: Mature pollen grain stage. (**A**) MDA contents, (**B**) SOD activities. Significant differences were assessed by Student’s *t*-test (** *p* < 0.01).

In this study, K-means clustering analysis of DEGs revealed that many ROS-related genes were clustered together, including ubiquinol-cytochrome-c reductase (Cluster 6), cytochrome-c oxidase (Cluster 1), F_1_F_0_-ATPase (Cluster 1), as well as those involved in the positive regulation of SOD (cluster 5), and the hydrogen peroxide catabolic and oxidation-reduction process (Cluster 11). Thus, to explore the relationship between abnormal pollen and ROS, we measured the MDA contents as well as the SOD activities of the anthers at different microspore developmental stages in 722HA and 722HB (Figure 6). The result showed that the MDA content in 722HA was higher than that in 722HB at all stages of microspore development and that there were significant differences between 722HA and 722HB at the mononuclear, dinuclear, and mature pollen grain stages (Figure 6A). Moreover, the MDA content continued to increase from the mononuclear stage onward in 722HA, while MDA content remained at a relatively stable level in 722HB (Figure 6A). In addition, the results showed that the SOD activity was consistently lower in 722HA than 722HB throughout microspore development and that there were significant differences between 722HA and 722HB at the four stages of microspore development (Figure 6B).

### 2.7. RT-qPCR Verification

**Figure 7:**
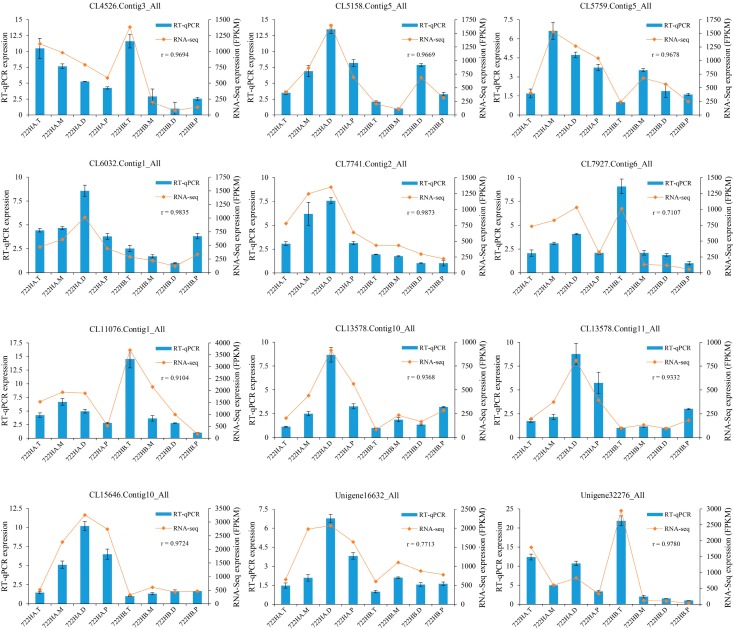
RT-qPCR verification results of 12 genes. The relative expressions levels of the twelve genes were calculated using the 2^−∆∆*C*t^ method. r: Represents the correlation coefficient between RT-qPCR data and RNA-seq data. 722HA.T, 722HA.M, 722HA.D, and 722HA.P correspond to microspores of the tetrad, mononuclear, dinuclear, and mature pollen grains stages, respectively, in 722HA; 722HB.T, 722HB.M, 722BA.D, and 722HB.P correspond to microspores of the tetrad, mononuclear, dinuclear, and mature pollen grain stages, respectively, in 722HB.

To verify the accuracy of our RNA-seq data, twelve genes were randomly selected, and their expression pattern was validated by RT-qPCR. The results showed that the expression patterns of these genes were essentially consistent with the expression patterns observed in the RNA-seq data, indicating that our RNA-seq data were reliable (Figure 7, Table S6).

## 3. Discussion

### 3.1. Premature Tapetum Degradation is Related to Pollen Abortion in 722HA

Microspore development is a critical biological process for reproductive success in plants. The tapetum plays a crucial role in microspore development and maturation by supplying necessary nutrients and structural components. Many studies have indicated that premature or delayed programmed cell death (PCD) by tapetal cells disrupts the supply of nutrients to microspores, thereby resulting in microspore/pollen abortion [15,26]. In the wheat CMS line U87B1-706A, delayed tapetal PCD and organelle disorder phenotype are present in the early mononuclear stage, and finally, shrunken microspores and disordered extracellular structure occur in the late mononuclear stage [27]. In the present study in kenaf, cytological observations revealed that pollen abortion was mainly due to nutrient deficiency caused by premature degradation of the tapetal cells in 722HA. Moreover, the contents of the pollen were sparse and plasmolysis at the dinuclear and mature pollen grain stages was observed (Figure 1C). On the basis of these results, the factors affecting pollen abortion in 722HA were consistent with those in other CMS systems, such as cabbage, radish, and rapeseed [1,28,29], suggesting that abnormal degradation of tapetal cells results in microspore/pollen abortion.

### 3.2. DEGs Involved in Electron Transfer Chain and F_1_F_0_-ATPase Proton Transport

Mitochondria provide sufficient energy for cells mainly through aerobic respiration, which involves a variety of metabolic pathways, including respiratory electron transport, ATP synthesis, the TCA cycle, glycolysis/gluconeogenesis [30]. In this study, K-means cluster analysis of DEGs revealed that the expression patterns of many genes related to mitochondrial respiratory chain (ubiquinol-cytochrome-c reductase, cytochrome-c oxidase) and ATP synthesis (F_1_F_0_-ATPase) significantly differed.

The mitochondrial electron transfer chain, which is a major site for ROS production, is located in the mitochondrial membrane and catalyses the transfer high-energy electrons from NADH/FADH to adjacent complexes [31]. Many studies have shown that when electron transfer is reduced, excess electrons directly combine with molecular oxygen to produce excess ROS [32,33]. In the present study, the expression levels of cytochrome-c oxidase (Complex IV) were lower from the tetrad stage to the dinuclear stage in 722HA (Cluster 1). In addition, ubiquinol-cytochrome-c reductase (Complex III) also exhibited sustained levels of low expression during the development of microspores in 722HA (Cluster 6). These results indicated that the low expression of these genes in 722HA lead to blockage of the electron transport chain, after which the electrons directly combined with oxygen molecules to produce excessive ROS.

F_1_F_0_-ATPase (Complex V) is an important component of mitochondria and is reversibly involved in the synthesis and hydrolysis of ATP, depending on the direction of an electrochemical gradient that is formed by the passage of protons [34,35]. Many studies have shown that mitochondria encoded subunits of the F_1_F_0_-ATPase are associated with male sterility in plants [36,37]. In wheat CMS line KTM3315A, downregulated expression of the genes whose products encode F_1_F_0_-ATPase slows down the electron transfer rate, and excess electrons combine with molecular oxygen to form ROS, thus initiating PCD in the anthers [33]. In the present study, K-means cluster analysis of DEGs revealed that the expression levels of these genes in 722HA were significantly lower from the tetrad stage to the dinuclear stage (Cluster 1). This result implies that the low expression levels of genes encoding subunits of the F_1_F_0_-ATPase in 722HA may affect the electron transport rate, leading to excess electron and ROS represent an accumulation.

Increasing evidence indicates that ROS are an important regulatory factor for cell growth, and their spatial distribution has an important influence on tapetum development in anthers [38,39]. When excess ROS accumulate, cells will be affected by oxidative stress, which leads to an increased electron transfer rate in the mitochondria, enhancing H_2_O_2_ production and ATP consumption, and even causing PCD [15,36,40,41]. MDA is usually considered an indicator of lipid peroxidation caused by ROS, and its contents can reflect the oxidative stress (i.e., ROS) level of the plant [42,43]. In the present study, biochemical analysis revealed that the MDA content in 722HA was higher than that in 722HB throughout the microspore developmental process. Moreover, the MDA content continued to increase from the mononuclear stage in 722HA, but the MDA content remained at a relatively stable level in 722HB (Figure 6A). These results revealed that the ROS content in 722HA was always higher than that in 722HB. ROS accumulation occurred from the mononuclear stage to the mature pollen grain stage in 722HA.

### 3.3. DEGs Involved in ROS Scavenging

To better resist oxidative stress (i.e., ROS), plant cells have highly efficient ROS-scavenging system and thereby maintain ROS homeostasis in their cells to reduce the effects of ROS on various biological macromolecules [31]. ROS scavenging depends on antioxidative enzymes, such as SOD and hydrogen peroxide catabolic and oxidation-reduction process [44]. In the present study, K-means clustering analysis of DEGs indicated that the expression level of genes related to positive regulation of SOD in 722HA was lower than that in 722HB at the three stages (tetrad, mononuclear, and dinuclear) of microspore development, and the expression pattern of these genes were gradually decreased with microspore development (Cluster 5). Thus, we analysed the activity of SOD at different microspores developmental stages. Our results showed SOD activities in 722HA were invariably lower than that in 722HB during microspores development. This result was similar to the gene expression trend (Figure 6B). In addition, the genes related to hydrogen peroxide catabolic and oxidation-reduction process, whose products represent another class of ROS scavenging enzymes, were clustered by K-means clustering analysis. In 722HA, the expression level of these genes at tetrad stage was significantly higher, but decreased dramatically with microspore development (Cluster 11). These results suggested that the gene expression levels of SOD and proteins related to hydrogen peroxide catabolic and oxidation-reduction processes were higher at the tetrad stage in 722HA, and therefore, the ROS level in the anther remained lower. However, the expression levels of these genes were significantly downregulated from the mononuclear stage to the mature pollen grain stage, so that ROS in the cells could not be removed promptly. This resulted in an intracellular ROS homeostasis that was disrupted due to excessive ROS accumulation.

### 3.4. ROS Accumulation May Cause Premature Tapetum Degradation

The anther development of the CMS line 722HA and its maintainer line 722HB exhibited the same expression pattern only at the tetrad microspore and then exhibited completely different developmental processes. During the tetrad stage and mature pollen grain stage, SOD activity was positively regulated in 722HB, SOD could remove excess ROS in a timely and efficient manner, followed by defence and extracellular secretion via the normal cell membrane and cell wall. In 722HA, the expression levels of genes encoding Complex III, Complex IV, and Complex V (ubiquinol-cytochrome-c reductase, cytochrome-c oxidase, and F_1_F_0_-ATPase) were dramatically changed from the mononuclear stage to the mature pollen grain stage, resulting in a reduced electron transfer rate and excess electrons combining with oxygen molecules to produce ROS. In addition, owing to the lack of positive regulation of SOD and the low efficiency of scavenging ROS by the hydrogen peroxide catabolic and oxidation-reduction processes, the intracellular ROS balance was disrupted, and premature PCD of tapetal cells occurred in the anthers. The contents of the pollen grains ultimately were sparse and plasmolysis because of the lack of nutrients at the later stage of development in 722HA.

## 4. Materials and Methods 

### 4.1. Plant Materials 

‘HMS’, a thermo-sensitive male-sterile mutant, was generated from a kenaf wild-type 722B by transforming the fragment of *HcPDIL5-2a* gene. The maintainer line 722HB was the previous generation inbred line. The cytoplasmic male-sterile line 722HA was obtained through repeated backcrossing (more than ten generations) of the male-sterile mutant ‘HMS’ and 722HB, of which 722HB was the recurrent parent. 722HA and 722HB had nearly identical nuclear genetic backgrounds, but their cytoplasm differed. Lines 722HA and 722HB were cultivated in the test field of Guangxi University (Nanning, Guangxi, China) under the same conditions. During the pollen development period, the kenaf CMS line 722HA flower buds with bud lengths (BLs) that were 2.5 ≤ BL < 3.5 mm, 3.5 ≤ BL < 5 mm, 5 ≤ BL < 7 mm, and BL ≥ 7 mm and the maintainer line 722HB flower buds with BLs that were 2.5 ≤ BL < 3.5 mm, 3.5 ≤ BL < 6 mm, 6 ≤ BL < 8 mm, and BL ≥ 8 mm, corresponding to the tetrad stage, mononuclear stage, dinuclear stage, and mature pollen grain stage [45], were individually collected on ice after the anthers were carefully removed. The kenaf anthers were then quickly isolated and frozen in liquid nitrogen, after which they were stored at −80 °C for extraction of total RNA and preparation for RNA-seq analysis.

### 4.2. Morphological and Cytological Observations

At the full-bloom stage, the flowers of the CMS line 722HA and its maintainer line 722HB were observed via a digital camera (Canon, Tokyo, Japan), and images of the flower buds were captured with a stereomicroscope (Olympus, Tokyo, Japan).

Flower buds of different lengths from 722HA and 722HB were vacuum-infiltrated and fixed in cold Carnoy’s fixative solution (ethanol:acetic acid = 3:1) for 24 h at 4 °C. The fixed floral buds were subsequently dehydrated though a graded ethanol series, up to 100% ethanol. The dehydrated floral buds were embedded in paraffin wax and subsequently sectioned to a thickness 10 µm. Serial sections of the floral bud tissues were mounted on glass slides and stained with pissophane-haematoxylin. The sectioned floral buds were observed and imaged via a DMI3000B microscope (Leica, Wetzlar, German).

The pollen of both lines was characterized by SEM, which was performed at Guangxi Medical University. Through a binocular dissecting microscope (Olympus, Tokyo, Japan), pollen was removed from each anther using a dissecting needle and placed on a metallic stub. For SEM observation, the samples were coated with gold using a JFC-15000 Ion Sputter manufactured by JEOL. The structures of the pollen grains were observed, and photomicrographs were captured by a Vega 3 LMU (Tescan, Brno, Czech Republic) scanning electron microscopes.

### 4.3. Total RNA Extraction, cDNA Library Construction, and Deep Sequencing

Total RNA for each sample was extracted via a Quick Plant RNA Isolation Kit (Huayueyang, Beijing, China) according to the manufacturer’s protocol. The RNA quality and quantity of each sample was then measured with a NanoDrop 2000 spectrophotometer (Thermo Scientific, Waltham, Massachusetts, USA). The RNA integrity was assessed by an Agilent 2100 Bioanalyzer (Agilent, Palo Alto, California, USA) in conjunction with an Agilent RNA 6000 Nano Kit. DNase I was used to degrade double-stranded and single-stranded DNA contaminants within the RNA samples. The RNA samples were ultimately divided into two groups for library construction and RNA-seq validation. A sequencing library for each RNA sample was prepared via the BGISEQ-500 transcriptome library workflow according to the manufacturer’s protocol (BGI, Shenzhen, China). Briefly, mRNA molecules were purified using oligo (dT)-attached magnetic beads. The mRNA was then fragmented into small pieces with divalent cations. With respect to the cDNA synthesis step, first-strand cDNA was synthesized via random hexamer-primed reverse transcription, followed by second-strand cDNA synthesis. The synthesized cDNA was subjected to end repair and then was 3′ adenylated. Adaptors were ligated to the ends of the 3′ adenylated cDNA, after which PCR amplification was performed, with the cDNA library used as a template. The PCR products were purified with solid phase reversible immobilization (SPRI) beads and then denatured by heat. The single-strand DNA was cyclized by splint oligo and DNA ligase. Each cDNA library (4–5 pM) was used for sequencing on a BGISEQ-500 platform, with 2 × 100 bp paired-end reads; original raw data (FASTQ format) were generated. The raw sequencing data of twenty-four samples from the four microspore development stages of both 722HA and 722HB were deposited in the NCBI Short Read Archive (SRA, accession number: PRJNA535382).

### 4.4. De Novo Assembly and Functional Annotation

To obtain clean reads, the raw reads in FASTQ format were filtered by removing the low-quality reads, which consisted of reads in which more than 20% of the bases had a quality lower than 10, reads containing adaptors, and reads with more than 5% unknown bases. The software Trinity (version 2.8.4, default k-mer = 25 bp) was used for the de novo assembly of the clean reads. Briefly, the de novo assembly process is as follows. Trinity software was first used to partition the clean reads into shorter k-mers and then extend them to form contigs. Contig clusters were then collected, after which de Bruijn graphs were constructed. The transcript sequences were ultimately obtained by combining linear paths with continuous nodes in the de Bruijn graphs. At the same time, the transcripts were clustered to generate unigenes by TIGR Gene Indices clustering tools (TGICL). At the unigene functional annotation step, Blastn (version 2.2.23) and Blastx (version 2.2.23) were used to align the unigenes to the nucleotide (NT), non-redundant nucleotide (NR), EuKaryotic Orthologous Groups (KOG), Kyoto Encyclopedia of Genes and Genomes (KEGG), and Swiss-Prot databases. Blast2GO (version 2.5.0) in conjunction with NR annotation was then used for Gene Ontology (GO) annotation, and InterProScan (version 5.11-51.0) was used for InterPro annotation. TransDecoder (version 3.0.1) (https://transdecoder.github.io) was used to identify the candidate coding regions of the unigene transcripts. The longest ORF (open reading frame) was extracted, after which the Pfam protein homologous sequences were searched by blast against Swiss-Prot and Hmmscan to predict the coding region. 

### 4.5. DEG Analysis

Clean reads were mapped to unigenes via Bowtie2 (version 2.2.5), and the FPKM values were then calculated for each unigene using RSEM (version 1.2.12). Differential expression analyses between 722HA and 722HB at different stages were performed by NOISeq. Genes meeting the following criteria were evaluated as DEGs: Probability ≥ 0.8 and |log_2_ (fold change)| ≥ 1. The DEGs were evaluated by hierarchical clustering using the pheatmap R package (version 1.0.12). PCA was performed with the R package PCA tools, and co-expression analysis was conducted using the k-means method in MeV software (V4.9). Hypergeometric tests of GO analysis and pathway functional enrichments of the DEGs were performed with the Stats R package (version 3.7.0). GO functional enrichment analysis and KEGG pathway functional enrichment analysis were performed via phyper, a function of R. The false discovery rate (FDR) for each *p* value was calculated; in general, the terms whose FDR < 0.01 were defined as significantly enriched. The Pearson and CLR methods of the R package minet were used to predict TF target genes with a screening threshold of ≥ 8. To identify TFs, the ORFs of each unigene were predicted using getorf and aligned to TF domains form PlnTFDB (http://plntfdb.bio.uni-potsdam.de) via hmmsearch.

### 4.6. Quantitative Real-Time PCR for RNA-seq Validation 

Total RNA samples at four stages (the tetrad, mononuclear, dinuclear, and mature pollen grain stages) of lines 722HA and 722HB were used to reverse-transcribe cDNA via HiScript II Q RT SuperMix for qPCR (+gDNA wiper) (Vazyme, Nanjing, China). The relative expression levels of twelve randomly selected DEGs were measured by RT-qPCR and calculated according to the 2^−∆∆*C*t^ method. To normalize the RNA expression levels of the samples, a combination of *TUB*, *CYP*, and *PEPKR1* were used as an internal reference gene [34]. Specific primers were designed with Primer 3 plus (http://www.primer3plus.com/). The primers used for RT-qPCR are listed in Table S7. RT-qPCR was conducted in a total volume of 15 µL that consisted of 7.5 µL of SYBR Green PCR master mix (TaKaRa, Dalian, China), 4.9 µL of RNase/DNase-free H_2_O, 0.5 µL of each primer, and 2 µL of cDNA. A CFX96 Real Time PCR System (Bio-Rad, Hercules, CA, USA) was used to conduct RT-qPCR, and the PCR program was as follows: 95 °C for 30 s, followed by 40 cycles of 95 °C for 15 s for denaturation and then 60 °C for 30 s for annealing. All RT-qPCR reactions were performed in triplicate technical replicates

### 4.7. Enzyme Activity and MDA Content Assays 

During the flowering period, anther samples from four stages of microspore development (tetrad, mononuclear, dinuclear, and mature pollen grain stage) in lines 722HA and 722HB were collected and used for physiological analysis. The anthers (0.5 g) were ground with 5 mL of precooled phosphate buffer (0.05 M, pH 7.8). The tissue homogenate was then centrifuged at 12,000× *g* at 4 °C for 10 min. The supernatant was collected and centrifuged again at 12,000× *g* at 4 °C for 10 min. The supernatant was ultimately collected and placed on ice to analyse the MDA content and SOD activity. The MDA content was measured according to previously published methods [32], and the SOD activity was also measured according to previously described methods [46]. Three biological repeats of all four microspore development stages were analysed for each material.

## 5. Conclusions

In this study, we conducted cytological, transcriptomic, and biochemical analyses between the kenaf CMS line 722HA and its maintainer line 722HB. The cytological observations revealed that, owing to the premature degradation of the tapetum at the mononuclear stage in 722HA, the microspores lacked nutrients at the late stage of development, the contents of the microspores were sparse, and cytoplasmic separation occurred. According to the transcriptome analysis, the expression pattern of many DEGs related to ROS metabolism were completely different. Biochemical analysis revealed that the capability of ROS scavenging decreased in 722HA, resulting in an accumulation of excess ROS. Our findings will help to elucidate the molecular mechanism governing pollen abortion in kenaf CMS line 722HA and provide a theoretical basis for better utilization of kenaf heterosis.

## Figures and Tables

**Table 1:** Results of de novo assembly.

Categories	Sub-Categories	722HA	722HB	Total
Reads	Total raw reads (M)	1230.85	1238.08	2468.93
	Clean reads (M)	1124.69	1129.13	2253.82
	Q20 (%)	97.75	97.64	97.69
	Q30 (%)	91.15	90.99	91.01
	Clean reads ratio (%)	91.34	91.22	91.2
Transcripts	Total number	1,410,176.00	1,310,102.00	2,720,278.00
	Total length (nt)	1,330,236,352.00	1,215,811,030.00	2,546,047,382.00
	Mean length (nt)	940	926.08	933.04
	N50 (nt)	1730.33	1679.00	1704.67
	GC (%)	41.83	42.05	41.94
Unigenes	Total number	1,011,304.00	937,700.00	1,949,004.00
	Total length (nt)	1,054,727,027.00	949,656,291.00	2,004,383,318.00
	Mean length (nt)	1039.00	1010.33	1024.67
	N50 (nt)	1781.17	1706.17	1743.67
	GC (%)	41.77	41.99	41.88

## Supplementary Materials

Supplementary materials can be found at https://www.mdpi.com/1422-0067/20/21/5515/s1. Table S1. Clean reads quality metrics. Table S2. Transcription factors annotate results. Table S3. GO enrichment (BP) of k-means clustering. Table S4. GO enrichment (MF) of k-means clustering. Table S5. A list of target genes corresponding to transcription factors. Table S6. Ct values at different stages of kenaf anthor in 722HA and 722HB. Table S7. Primers sequences and their product size in RT-qPCR. Figure S1. Gene ontology classification of all the transcripts in 722HA and 722HB. Figure S2. Gene expression distribution (FPKM) of each sample. Figure S3. Heat map of sample correlation based on gene expression profile.

## Abbreviations

CMS	Cytoplasmic male sterility
ROS	Reactive oxygen species
DEGs	Differentially expressed genes
SOD	Superoxide dismutase
MDA	Malondialdehyde
