# Supplementary material for: Comparative Transcriptome Analysis Reveals the Cause for Accumulation of Reactive Oxygen Species During Pollen Abortion in Cytoplasmic Male-Sterile Kenaf Line 722HA
Source: Int J Mol Sci. 2019 Nov 5;20(21):5515. doi: 10.3390/ijms20215515 (PMC6862637; doi:10.3390/ijms20215515)
Supplement: Supplementary file 1 [file ijms-20-05515-s001.zip › Supplementary materials/Supplementary Table title and Figure legend.docx]

**Table S1.** Clean reads quality metrics.

**Table S2.** Transcription factors annotate results.

**Table S3.** GO enrichment(BP) of k-means clustering.

**Table S4.** GO enrichment(MF) of k-means clustering.

**Table S5.** A list of target genes corresponding to transcription factors.

**Table S6.** Ct values at different stages of kenaf anthor in 722HA and 722HB.

**Table S7.** Primers sequences and their product size in RT-qPCR.

**Figure S1.** Gene ontology classification of all the transcripts in 722HA and 722HB.

**Figure S2.** Gene expression distribution (FPKM) of each sample. 722HA.T, 722HA.M, 722HA.D, and 722HA.P correspond to microspores of the tetrad, mononuclear, dinuclear, and mature pollen grains stages, respectively, in 722HA; 722HB.T, 722HB.M, 722BA.D, and 722HB.P correspond to microspores of the tetrad, mononuclear, dinuclear, and mature pollen grain stages, respectively, in 722HB.

**Figure S3.** Heat map of sample correlation based on gene expression profile. 722HA.T, 722HA.M, 722HA.D, and 722HA.P correspond to microspores of the tetrad, mononuclear, dinuclear, and mature pollen grains stages, respectively, in 722HA; 722HB.T, 722HB.M, 722BA.D, and 722HB.P correspond to microspores of the tetrad, mononuclear, dinuclear, and mature pollen grain stages, respectively, in 722HB.
