# Supplementary figures and images for: Comparative Transcriptome Analysis Reveals the Cause for Accumulation of Reactive Oxygen Species During Pollen Abortion in Cytoplasmic Male-Sterile Kenaf Line 722HA
Source: Int J Mol Sci. 2019 Nov 5;20(21):5515. doi: 10.3390/ijms20215515 (PMC6862637; doi:10.3390/ijms20215515)

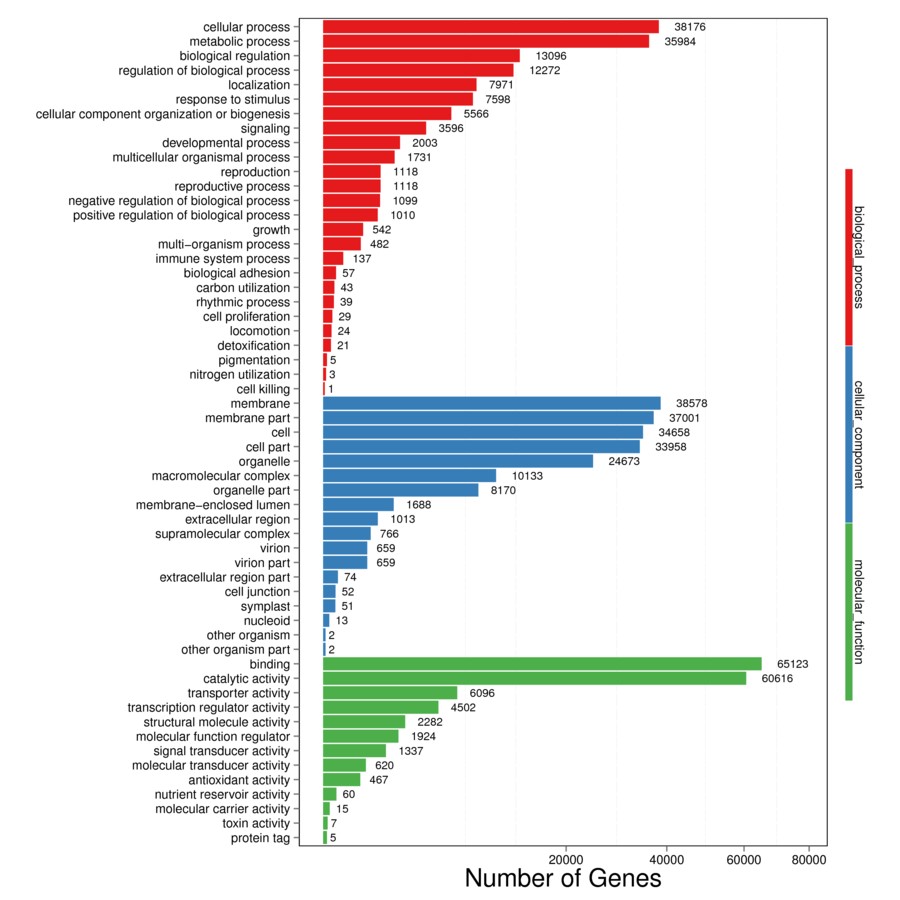

Supplement: Supplementary file 1 [file ijms-20-05515-s001.zip › Supplementary materials/Supplementary Figure S1.jpg]

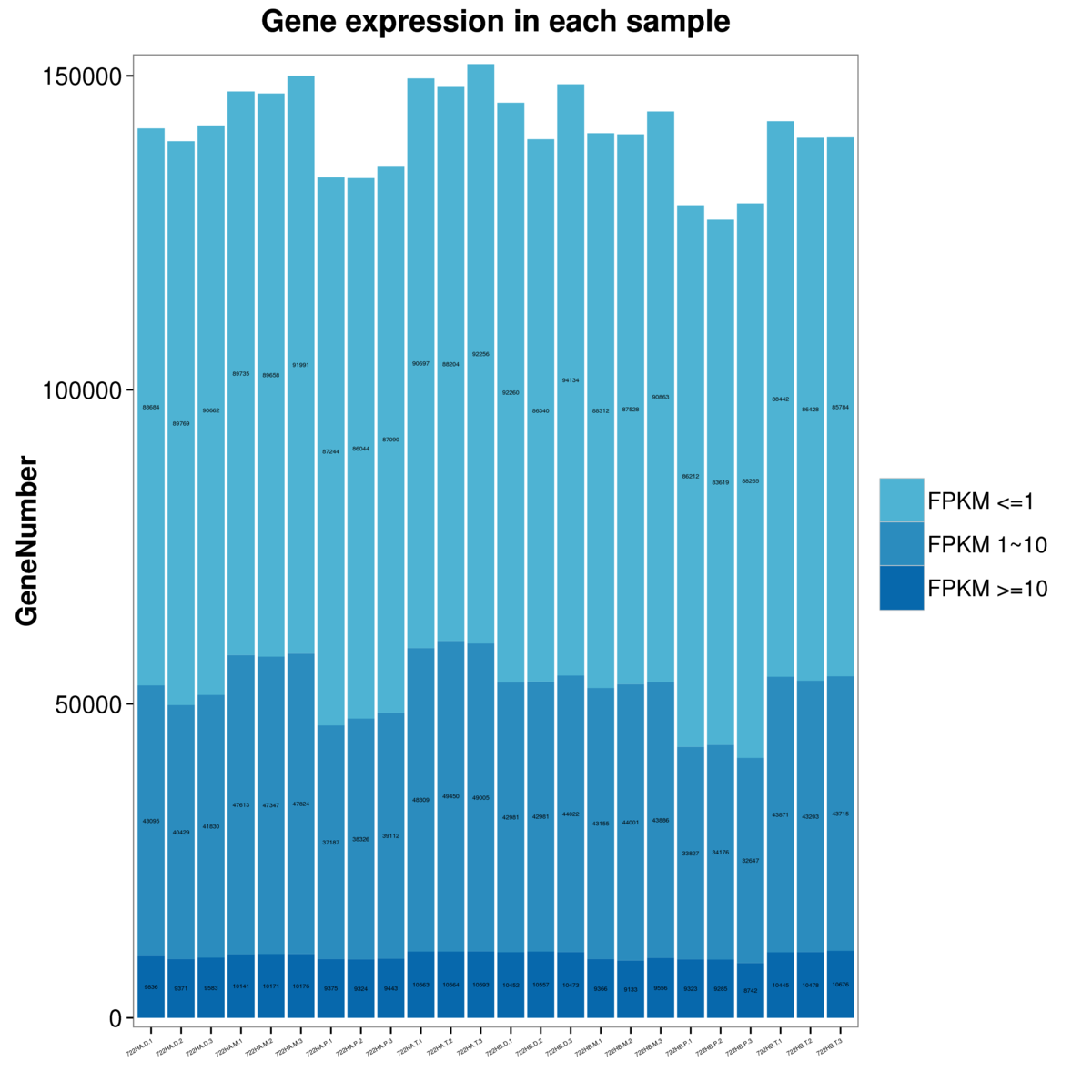

Supplement: Supplementary file 1 [file ijms-20-05515-s001.zip › Supplementary materials/Supplementary Figure S2.png]

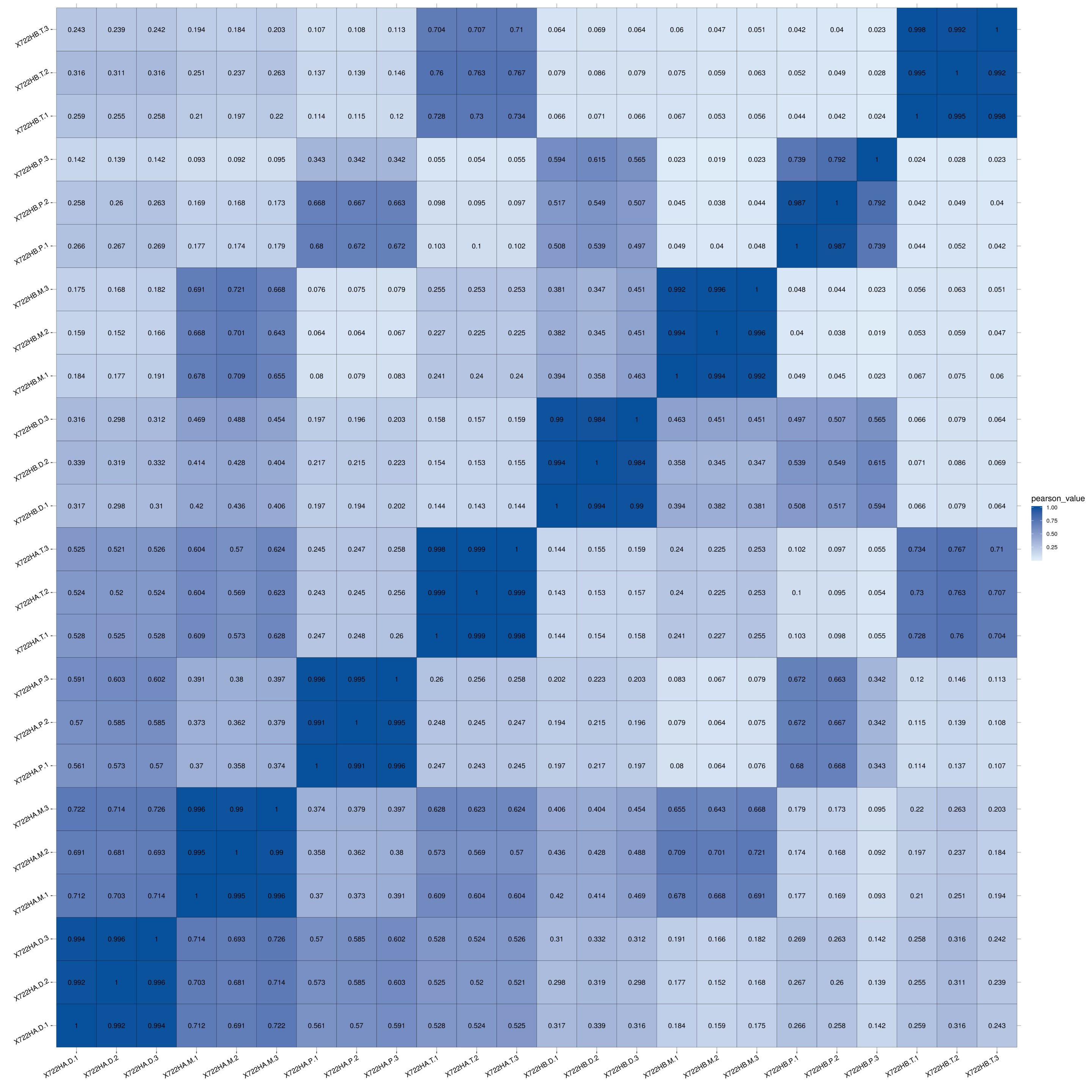

Supplement: Supplementary file 1 [file ijms-20-05515-s001.zip › Supplementary materials/Supplementary Figure S3.jpg]
